# The Effect of Random Error on Diagnostic Accuracy Illustrated with the Anthropometric Diagnosis of Malnutrition

**DOI:** 10.1371/journal.pone.0168585

**Published:** 2016-12-28

**Authors:** Emmanuel Grellety, Michael H. Golden

**Affiliations:** 1 Research Center Health Policy and Systems - International Health, School of Public Health, Université Libre de Bruxelles, Brussels, Belgium; 2 Department of Medicine and Therapeutics, University of Aberdeen, Aberdeen, Scotland; Hunter College, UNITED STATES

## Abstract

**Background:**

It is often thought that random measurement error has a minor effect upon the results of an epidemiological survey. Theoretically, errors of measurement should always increase the spread of a distribution. Defining an illness by having a measurement outside an established healthy range will lead to an inflated prevalence of that condition if there are measurement errors.

**Methods and results:**

A Monte Carlo simulation was conducted of anthropometric assessment of children with malnutrition. Random errors of increasing magnitude were imposed upon the populations and showed that there was an increase in the standard deviation with each of the errors that became exponentially greater with the magnitude of the error. The potential magnitude of the resulting error of reported prevalence of malnutrition were compared with published international data and found to be of sufficient magnitude to make a number of surveys and the numerous reports and analyses that used these data unreliable.

**Conclusions:**

The effect of random error in public health surveys and the data upon which diagnostic cut-off points are derived to define “health” has been underestimated. Even quite modest random errors can more than double the reported prevalence of conditions such as malnutrition. Increasing sample size does not address this problem, and may even result in less accurate estimates. More attention needs to be paid to the selection, calibration and maintenance of instruments, measurer selection, training & supervision, routine estimation of the likely magnitude of errors using standardization tests, use of statistical likelihood of error to exclude data from analysis and full reporting of these procedures in order to judge the reliability of survey reports.

## Introduction

For many illnesses biochemical, anthropometric or other measurements are made and compared with the distribution of the parameter derived from a normal healthy population. In order to assess the prevalence of the illness in the population an epidemiological survey is conducted and the proportion of the survey population that falls outside the “healthy” range is defined as having the condition. Measurements are taken from sufficient subjects to assess the confidence intervals of the prevalence using standard epidemiological statistical techniques.

It is usually assumed that increasing the number of subjects in the survey will automatically improve the precision of the assessment, and standard formulae are applied to determine the optimum number of measurements, the sample size, that need to be made to achieve the desired precision. It is often assumed that the effect of random errors in the measurements will be neutral because those erroneous measurements which overestimate will be matched by those that underestimate the parameter measured and thus effectively cancel each other out. For this reason most survey guidelines relegate the possible effects of random error to having a minor effect at best and the emphasis is placed on the sampling frame and increasing the sample size to improve the precision, and thus the assumed accuracy, of the estimated parameter. However, increasing the sample size may also lead to deterioration in the care that is taken with each measurement, particularly if this involves increasing the number of observers, less practice, poorer supervision or training and using multiple centres.

The prevalence of “cases” depends upon the number of measurements falling outside the normal range and thus into the tails of the distribution; that is below or above a cut-off point. We have therefore examined the effect of random errors of measurement on the numbers of cases falling outside the normal range that defines the illness; for this we have used the example of the anthropometric assessment of malnutrition in children.

### Theoretical considerations

**Fig 1:**
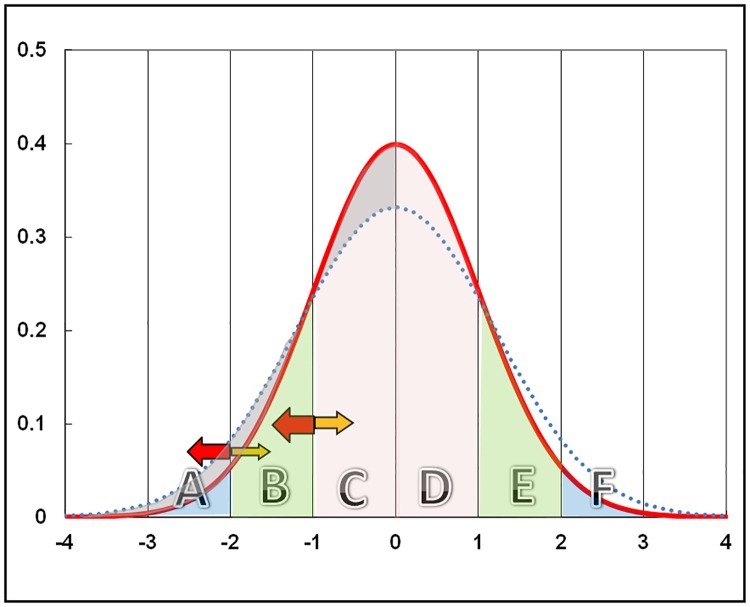
Effect of random error on a Gaussian distribution. Gaussian distribution curve illustrating how a random error in measurement will result in different proportions of data-points moving from one segment to another to increase the value of the standard deviation. The red line has a Standard deviation of 1.0. The dotted blue line has a standard deviation of 1.2. The grey areas show how the area in the centre of the curve has moved towards the tails thus increasing the areas below -2.0Z and above 2.0Z. Both distributions are Gaussian without generating moments of kurtosis or skewness. For explanation of the letters see the text.

Consider Fig 1, which shows a normal (Gaussian) distribution with vertical gridlines at one standard deviation intervals. If there is a random (non-systematic) error in a measurement then the recorded value will either move to the right or left of the true value with respect to the other values in the distribution, and this movement of data points will be entirely at random.

Consider the points recorded in area C. With random error some of them will move into area D and some into area B. Similarly, some of the points in D will move into C and others into E, etc. With a Gaussian distribution, the number of points entering C from D should be balanced by the points moving from D to C so that the mean value will not change with random error. This is the assumption that is tacitly made and the reason why random error is frequently not considered to be an important source of error. It is also the reason why many epidemiologists consider that the precision of an estimate is always improved by simply increasing the sample size, even if this involves using less experienced staff with consequent deterioration in quality control.

In contrast, the number of points moving from C to B is a function of the area of C and the number of points moving from B back to C a function of the area of B. These areas are not the same. Thus, with a random error in measurement there will be more points moving from C to B than from B to C. Similarly there will be more points moving from B to A than from A to B. The same effect will be seen at the upper end of the distribution so that more points will move from D to E than from E to D and more from E to F than from F to E so that symmetry is maintained and random error should not generate moments of skewness or kurtosis. The resulting effect of a random error will simply be to broaden the distribution. If the distribution is Gaussian then the results of random error will be neutral in terms of the mean and the distribution will remain Gaussian, but there will be an increase in the variance and thus the standard deviation (SD) of the distribution. The magnitude of the effect will depend upon the mean magnitude of the errors of measurement. If the errors themselves are random and are plotted they are also expected to form a distribution with a mean of zero and a standard deviation related to the absolute size of the errors; if this were not the case then the errors would have a systematic bias component and not be entirely random. Theoretically random errors always increase the SD, never diminish the SD. These errors will presumably be related to the technical error of the measurement (TEM) which should be determined before, during and after a series of measurements in a survey [1,2]. The observed variance (SD^2^) will then be related to the true variance of the variable being measured plus the variance of the errors (TEM^2^) of the measurers, and a better estimate of the true prevalence could be statistically computed by subtraction of the variance of the TEM from the observed variance of the measurements.

The effect that this has on the proportion of a distribution falling outside the cut-off point being used to define abnormality, in relation to the true proportion outside this cut-off point is then a function of the relative areas of the segments above and below the cut-off point chosen. Because the ratio of adjacent segments increases the further they deviate from the mean value the effect will be increasingly pronounced as the tails of the distribution are approached and the further the cut-off point is from the mean the greater the effect of a random error on the observed prevalence. Table 1 gives the relative areas of the segments shown in Fig 1. Thus, 2.5 more measurements will move from the 0/1 SD segment (C, D) than will move from the 1/2 segment (B, E) back to the 0/1 segment. At the tails of the distribution, for every one measurement that moves from the 3/4 SD segment to the 2/3 SD segment, 16.2 points will move from the 2/3 SD segment to the 3/4 SD segment. The effect will affect both the upper and lower tails of the distribution.

**Table 1:** The relative movement of points with distance from the mean.

SD segment	marked	Area	relative to the area above
0 to 1	C & D	0.341	1.0
1 to 2	B & E	0.136	x 2.5
2 to 3	A & F	0.021	x 6.4
3 to 4	tail	0.001	x 16.2
4 to 5	tail	3^-5	x 42.0
<5	tail	3^-7	x 109.5

It is usually the case that an illness is diagnosed when an individual falls beyond the distribution seen in 95% of a “healthy” population (i.e. ±2 SD with 2.5% of “unhealthy” below, and 2.5% above the cut-off) 5% will be classified as “unhealthy”. But with such conditions as acute malnutrition, obesity, anaemia, hypertension, diabetes, etc. random measurement error has the potential of inflating the numbers diagnosed as unhealthy far beyond this 5%. Where a more extreme cut off point is used such as ±3 SD only 0.135% of truly “healthy” subjects will fall below/above this cut-off. The effect of measurement error can then start to dominate estimates, so that the majority of values recorded outside this range are more likely to be erroneous values than true “cases”. The ratio of true to false values below the cut-off point will then be a function of the population mean, the cut-off point and the magnitude of the random error (the overall TEM of the measurers). The reported prevalence will not only depend upon the degree to which the mean and distribution of the observed population deviates from the mean and distribution of the healthy population, but also on the precision with which the measurements have been made. Importantly, this effect is completely independent of the sample size; it depends only on the precision with which the measurements are taken.

### Effect of measurement error on anthropometric variables used to assess malnutrition in children

Anthropometric surveys of children are conducted to assess the nutritional status of a population [3]. Weight, height, and mid-upper arm circumference (MUAC) are the primary measurements taken; these are standardised for age & sex, combined and compared with international standards, in this case the WHO_2006_ standards [4] to determine the individual’s weight-for-height Z-score (WHZ), height-for-age Z-score (HAZ), weight-for-age Z-score (WAZ) and MUAC-for-age (MUACage) or absolute MUAC. Each measurement is always subject to measurement error [5–7] and compounded when entered into the equations using more than a single measurement. The precision of the instruments used and the published standards’ intervals often requires height to be rounded to the nearest 1.0 or 0.5 cm, weight to the nearest 100g, age to the nearest month and MUAC to the nearest 1mm; these approximations are not thought to have more than a trivial effect on the results [8].

There is a difference of height of about 0.7cm between measuring a child recumbent and standing [9]. There is also a mean diurnal variation in height of about 0.7cm in children less than 5 years of age [10]; this depends upon the length of time that the child has been standing, running, playing and otherwise active. This variation is thought to be due to compression of the inter-vertebral discs [11]. Children measured early in the morning are likely to be different from those measured late in the day; malnourished children are likely to be less active than well-nourished children. Measurements made in paediatric practice in the USA showed a mean difference of 1.2cm ± 1.6cm (SD) between the practitioners and experienced nurses specifically trained in anthropometry; only 30% of the measurements were within 0.5cm [12].

Although, the scales used in surveys have divisions of 100g the children frequently move whilst being weighed so that the needle may deviate by several divisions. Frequently, nappies, socks or pants are not removed; the amount of food, stool, urine and the state of hydration of the child are other sources of variation.

Age is the most problematic of the variables. In most Western cultures it is rounded down to the nearest completed month, in other cultures age is rounded up to the next highest month. In most developing countries there is no birth registration and birthdays are not celebrated; consequently, as a child ages the actual age becomes increasingly vague in the memory of the mother. Age is then approximated by using a calendar of local memorable events and the mother asked to remember to which event the birth most closely approximated. These calendars are normally quite crude and are not capable of identifying the actual month of birth. The questioning takes some time and is often administered in a perfunctory way, particularly if the survey team is tired or asked to complete excessive interviews in a single day. Even with full birth registration, there are often quite gross errors in reported age in young children; in a census from Scotland 7.7% of ages were erroneously reported by the respondent [13]. In Singapore children’s age in census data of respondents giving ages in English showed that 66% were erroneous [14]. In Ghana age was misstated in 35% of children by a year or more [15]; similarly, in Bangladesh, 26% of under 6 year old children had their ages misstated by a year or more [16], and the error increases with age up to 5 years [17,18]. Even when there is a birth registration paper, this may be grossly in error. Delayed registration is the usual cause for error [19]. In Mali children must be registered within 7 days of birth, if this date is missed then the parent has to attend court in a distant administrative centre to obtain a birth paper. This results in children being registered as being born within 7 days of the date of registration, even if the child was born long before that date [20]. There can also be problems with calendars and customs; for example, the Chinese traditionally record a child as one year at birth and this is incremented by one year as each Chinese new year passes—traditional Chinese may state that a child is two years of age shortly after birth if there happens to be a New Year between the birth and the survey [21]. Of more concern is an interaction with nutritional status and misstatement of age, with errors most frequently found in the malnourished [22].

The MUAC tape is a narrow plastic band placed around the upper arm; the reading obtained depends critically on the tension that the enumerator applies to the tape when the reading is taken. Experience shows that even with trained experienced observers it is rare to get a technical error of the measurement of less than 2mm.

To these errors must be added errors due to digit preference, number transposition and recording & data entry [23,24]. It is common practice for surveys in developing countries for survey teams to be recruited from the friends and families of those involved in the survey and they usually have almost no experience; the team members’ eye sight is not usually checked and glasses are expensive and uncommon in many countries.

In anthropological research and in collection of data to set standards the investigators take great care to minimise measurement errors [11,25] and estimates of the errors are made and reported, but this is not usually the case when surveys are carried out for other purposes. Some errors are systematic and epidemiologists take great care to try to eliminate systematic errors; but there are always random errors. Studies only vary in the degree of those errors which are generally thought to be reduced or eliminated by simply increasing the sample size.

In order to investigate the effect of random errors on the derived prevalence we generated artificial populations of known distribution and then imposed random errors on the data and examined the effect on the distribution and the prevalence of malnutrition recorded.

## Methods

### Monte Carlo simulation of populations containing malnourished children

To examine weight variation, 50 populations, each of 2020 subjects were generated. These were composed of 10 boys and 10 girls at each 0.5 cm interval in height from 60 to 110 cm (101 intervals). To examine age related variables (height-for-age and MUAC-for-age) the populations generated were of 2160 subjects composed of 20 boys and 20 girls aged at each month from 6 to 59 months (54 intervals).

In order to generate the simulated populations, for the anthropometric variable under consideration, the LMS (lambda-mu-sigma) parameters were downloaded from WHO’s website [26] and entered into an Excel spread sheet corresponding to each sex specific height (for weight analysis) and age (for height and MUAC analysis) of the child in the reference population. To generate a population of children with a given distribution for WHZ, each child’s weight was generated using the following equation:

Weight = M x ((Z x S x L) +1)^(1/L)  (1)

Where LMS are the parameters that define the sex and height specific distribution of each individual; Z is the normally distributed Z-score that has been randomly assigned to the child with a defined mean and standard deviation (most of the population’s mean Z-score was either -0.6 Z or -1.0 Z: all simulated populations were generated with a standard deviation of 1.0 Z-score).

For example, the WHZ parameters for a 70.0 cm male are L = -0.3521; M = 8.4227; S = 0.08229. To obtain the weight corresponding to a WHZ of -2.7 Z that could have been assigned to this individual child the equation is solved as follows:

Wt(Kg) = 8.4227 x ((−2.7 x 0.08229 x −0.3521)+1)^(1/−0.3521) = 6.801kg

In this way populations were generated with a uniform distribution of height (60 to 110 cm) or age (6 to 59 months) and an equal number of males and females. The height and age distributions of the simulated populations were not adjusted to allow for death of children during these intervals or for variations in birth rate and growth of the population. No attempt was made to examine subgroups of children’s height or age to focus on those ages which experience a higher or lower prevalence of malnutrition; the randomly assigned Z-scores were thus evenly distributed across the age and height intervals of the simulated populations.

To examine the effect of error in the WHZ, HAZ and MUAC/age random errors in the weight, height, age and MUAC were generated using the Mersenne Twister algorithm [27] in PopTools [28]. The random errors were then added to the “true” values of the simulated populations and compared with the distribution of the same population before the random errors were added. The added random errors all had a Gaussian distribution with a mean of zero. The standard deviation of the random errors was varied systematically to examine the effect of the magnitude of the error on the distribution of the anthropometric variable. The number of children that fall below the cut-off points for global acute malnutrition (GAM = moderate acute malnutrition + severe acute malnutrition), defined as <-2.0 Z or MUAC of <125 mm and the number that fall below the cut-off points for severe acute malnutrition (SAM) defined as <-3.0 Z or a MUAC of <115 mm were counted. Similar analyses were performed with height-for-age to determine the effects of errors in height and age in the prevalence of stunting in height.

For each magnitude of the error, replicated error-populations were generated by adding the generated random error in weight to the “true” weight of the reference-population. The resulting Z-score of the children with the newly generated erroneous weights were then obtained using the formula:

WHZ = ((Wt/M)^L−1)/(L x S) (2)

Using the example above let us consider that the random error in one trial added to the weight of the 70 cm male child was +158g. The erroneous weight would then be 6.801 + 0.158kg = 6.959Kg. We solve the equation:

WHZ = ((6.959/8.4227)^−0.3521 −1)/(−0.3521 x 0.08229) = −2.4 Z.

Thus, we can conclude that, for this child, an error of plus 158 g has changed the calculated Z-score from -2.7 Z to -2.4 Z. Similarly if that child had been assigned an error of minus 204g then his erroneous Z score would have been -3.1 Z and he would have moved from having a diagnosis of moderate acute malnutrition to one of severe acute malnutrition.

To examine the effects of an error in both height and in weight the same procedure was used to impose an error in weight, and a similar independent procedure was used to impose an error in the height of the individual children. As there were no intermediate LMS values between the 0.5 cm intervals for the heights of the children these were interpolated. The data from the 2020 children were individually pasted into the data entry spreadsheet of ENA for SMART software [29] (which has an interpolation algorithm incorporated to obtain values for children in intermediate heights) and the resultant WFH Z-scores generated.

A modification was used to investigate the effects of an error in age. Because as a child gets older there is an increasing error in the reported age so that the age-error in a 6 month old child is likely to be much less than the age-error in a 5 year old child [17,18,30], the normally distributed random error imposed upon the age was multiplied by the true age divided by 12, before adding the age-error to the true age, using the formula:

error x child’s age / 12 (3)

All units are in months; “error” is the random error of age. Thus, for example, where the random number generator assigned an error of 1.20 months, this magnitude of error would only have been added specifically to a child of exactly 12 months of age. The age-error added to the true age of a 6 month old child would be 0.60 months, whereas, for a 4 year old child the added age-error would be 4.80 months (1.2 x 48/12 = 4.8). The height-for-age data sets were used to determine the effect of an error in age on the derived estimates of stunting prevalence.

The effect of rounding was explored by dividing the weight by 1…n rounding the weight to the nearest 0.1 Kg and then multiplying the weight by 1…n to return the weight to its original magnitude rounded to “n” to emulate the effects of digit preference on the resulting Z-score.

### Examination of the WHO database

To examine the potential effect of measurement errors on published data, the prevalence of children having a weight-for-height of <-2 Z and <-3 Z were downloaded from the WHO global data base of malnutrition [31] using the NCHS references and not the new WHO_2006_ data; nevertheless, these are used to illustrate the potential problems of error on estimates of country specific malnutrition. With a Gaussian distribution there is a unique mean and SD for every particular percentage of children <-2 Z and <-3 Z; i.e. there is a known relationship between the proportion of the population with GAM and SAM. The relationship between the proportions of children with WHZ and HAZ below -2Z and -3Z were plotted and compared graphically to the theoretical relationships that would obtain if the SD of the survey had been 0.8, 1.0 and 1.2.

## Results

### Weight-for-height with weight measurement error

To examine the effect of variation in the magnitude of error in weight on weight-for-height assessment 50 separate populations were generated, each with a mean WHZ if -0.6 Z and a standard deviation of about 1.0 Z (95%CI, 1.0008, 0.9992). Each survey had 50 replicate random errors imposed at each 100g interval (the maximum precision of the scales used in surveys) from 0 to 1000g. Thus, for each error 2500 results were considered (50 replicates of 50 individual surveys).

**Fig 2:**
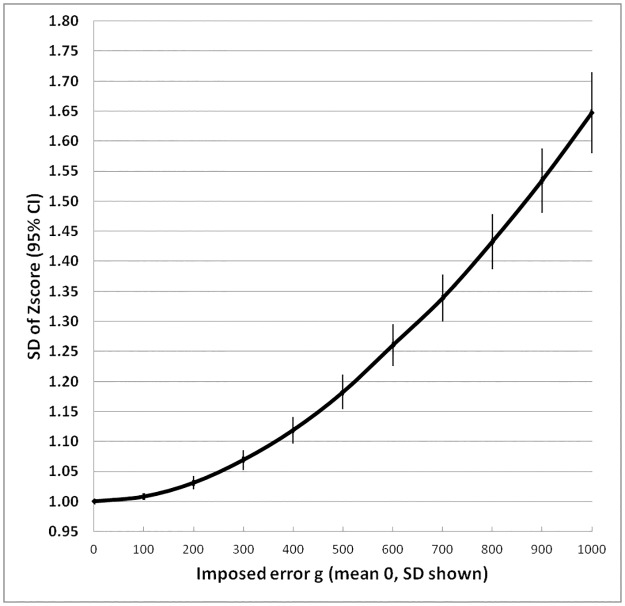
Effect of imposed random error in weight on SD of the distribution of weight-for-height Z scores. The error bars are 95% confidence intervals. All results use WHO_2006_ standards.

**Fig 3:**
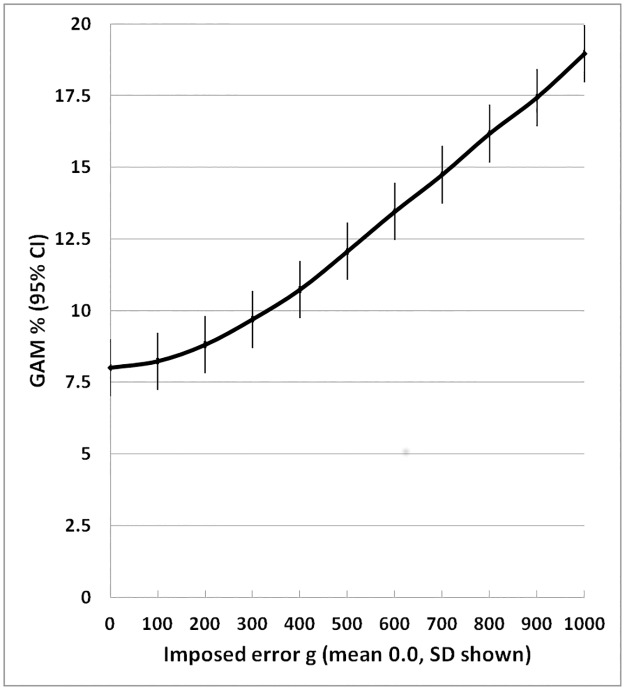
Effect of imposed random error in weight on prevalence of global acute malnutrition (weight-for-height <-2 Z). The error bars are 95% confidence intervals.

**Fig 4:**
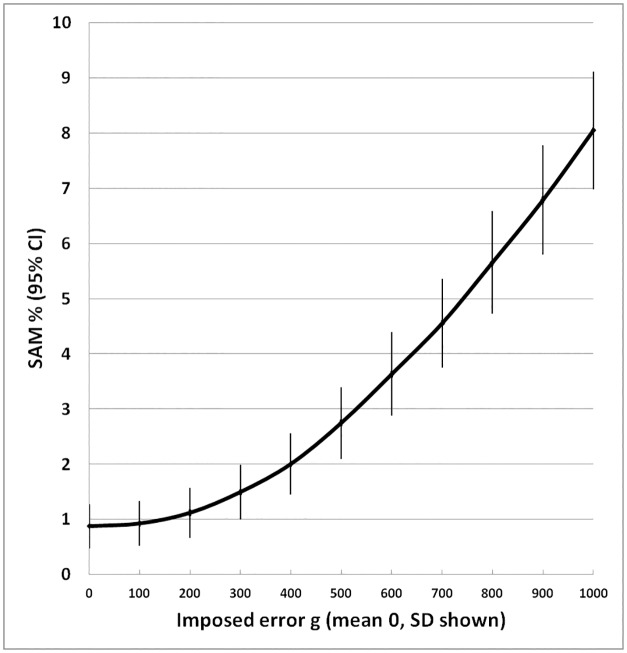
Effect of imposed random error in weight on prevalence of severe acute malnutrition (weight-for-height <-3 Z). The error bars are 95% confidence intervals.

The results of the errors in weight on the standard deviation, and prevalence of GAM and SAM of surveys with a mean of -0.6 Z and a standard deviation of 1.0 Z are shown in Table 2 and illustrated in Figs 2 to 4. With each increase in the magnitude of the random error there is an increase in the width of the standard deviation of the distribution of Z-scores so that more and more children would be classified as having global acute malnutrition. The level at which WHO declares a “severe nutritional situation” is a prevalence of 10% GAM; >15% is described as “critical” demanding immediate intervention [32]. In the present example where the true GAM was 8% a severe situation would be declared if there had been a random error of between 300 and 400g in weight measurement (Fig 3); if there was also an accompanying error in height measurement then the random error in weight would have needed to be much smaller to change the category of this population from “acceptable” to an “severe”.

**Table 2:** Effect of imposed random error in weight on distribution of weight-for-height.

SD of error	SD-Z	GAM	SAM
g	Z	%	%
0	1.000	8.01	0.87
100	1.008	8.23	0.92
200	1.031	8.81	1.12
300	1.069	9.70	1.49
400	1.119	10.73	2.00
500	1.182	12.06	2.75
600	1.260	13.45	3.63
700	1.338	14.74	4.55
800	1.432	16.17	5.66
900	1.535	17.43	6.78
1000	1.647	18.96	8.05

Fifty replicates of 50 surveys, each with a mean of -0.6 Z and SD of 1.0 Z-score were generated and random error added to each survey. SDs of over 1.1 are shaded in light pink and those over 1.2 in pink.

The effect on severe acute malnutrition is even more pronounced (Fig 4) with an increase from less than 1% SAM to over 9% with the largest error investigated.

**Fig 5:**
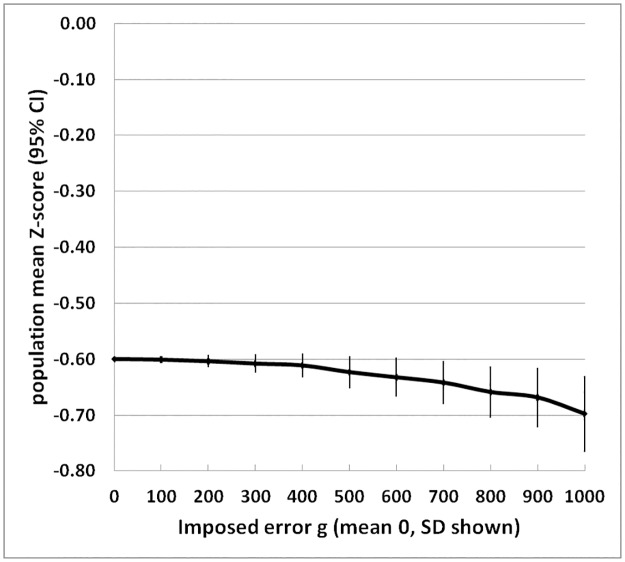
Change in the population mean Z-score from 50 simulated surveys with an initial mean of -0.6 Z, with imposed measurement errors in weight. Error bars show 95% confidence intervals.

During the imposition of the errors on the distribution, unexpectedly, it was noticed that there was a change in the mean value of the 50 test population surveys. This is shown in Fig 5. When the mean Z-score is negative, a further decrease in the mean Z-score of the population with imposed error will increase the observed or calculated prevalence of SAM and GAM in addition to the effect of an increase in the standard deviation of populations’ WHZ distribution. This effect on the mean, is generated from the fact that the WHO_2006_ “Z-scores” are not actual multiples of the SDs of the population distribution of the 6 reference populations used by WHO to generate the 2006 standards, rather they are derived from the centiles of the population’s distribution and converted to so called “Z-scores” where the centile would fall on a normal distribution. Thus, the WHO’s “Z-scores” are not what is normally conceived as a Z-score in the statistical literature because the weight differences between 0 Z and -1 Z, -1 Z and -2 Z, -2 Z and -3 Z, etc. are all different. Thus, an imposed error has less effect upon the resulting change in WHZ if the error falls on a child near the median than if it falls on a child further from the median; as the population mean becomes more negative, an imposed positive error will have less effect on the distribution than an imposed negative error. The effect is relatively minor compared to the effect of increasing the magnitude of the error itself. However, the assumption, when using the WHO_2006_ standards, that the mean value is not affected by a random measurement error is not correct. Thus, measurement error imposed in these circumstances has a slightly greater effect upon the calculated prevalence than would be the case with an increase in the width of the distribution alone.

### Effect of measurement error on the distribution of WHZ with different population mean WHZ

**Fig 6:**
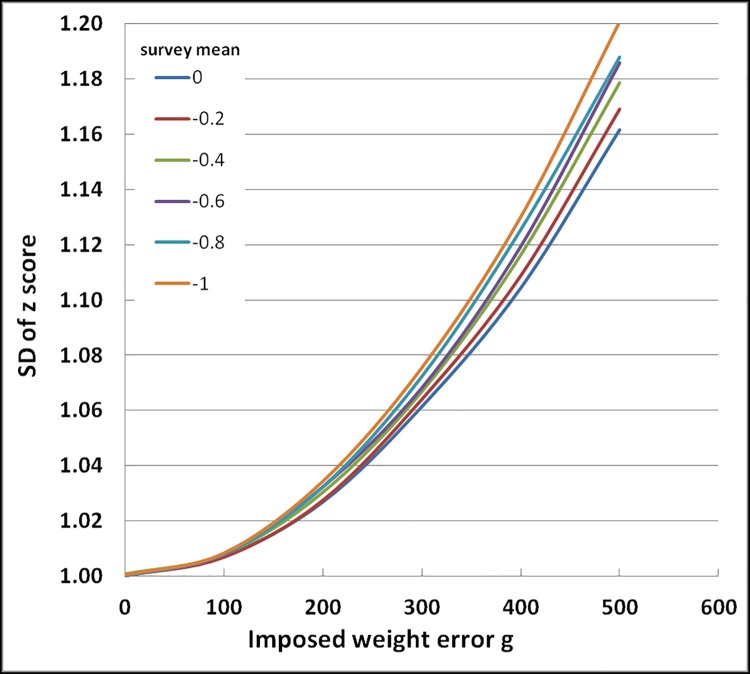
Effect of random error in weight, with no error in height, on the Change in SD of the WHZ distribution from 6 simulated surveys each with different population mean WHZ–Z-scores. The population mean WHZs were 0,-0.2,-0.4,-0.6,-0.8 and -1.0 Z.

**Fig 7:**
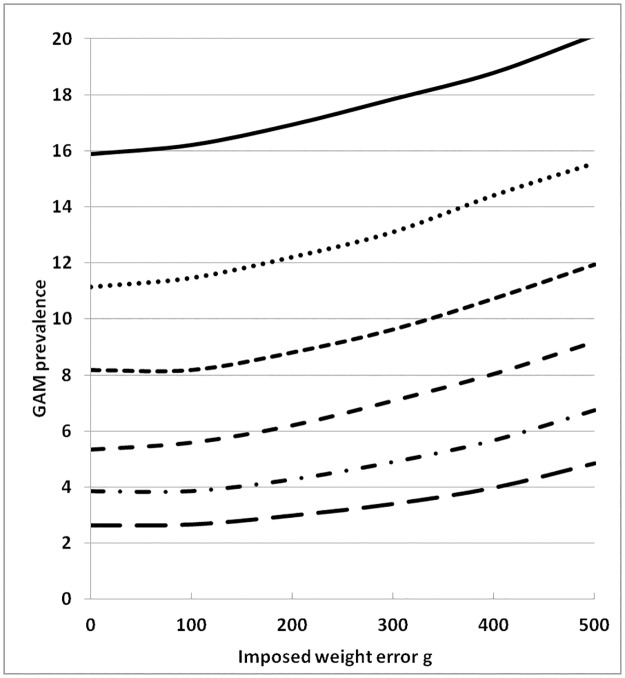
Effect of random error in weight, with no error in height, on the Change in GAM prevalence from 6 simulated surveys each with different population mean WHZ–Z-scores. The population means were 0 (bottom dashed line),-0.2,-0.4,-0.6,-0.8 and -1.0 Z (top solid line).

In view of the change in the mean value with imposed error, weight-for-height surveys were constructed with means ranging from 0.0 Z to -1.0 Z. The SDs of the distributions are shown in Fig 6 and the corresponding changes in GAM in Fig 7. There is an increase in the SD with the imposition of error as before; however, the rate of increase of the SD becomes greater as the mean of the distribution deviates progressively from 0.0 Z. This would not happen if the distribution of the WHO standard was truly Gaussian and is a consequence of slight difference in actual weight changes in the intervals 0.0 to -1.0Z, -1.0Z to -2.0Z etc. of the standards. Thus, with the WHO_2006_ standards as a population becomes more malnourished the effect of measurement error is exaggerated. Nevertheless, the changes in SD with changing mean values for the population WHZ Z-scores is relatively small compared to the change that occurs with increasing error of the measurement.

### Effect of “rounding” and digit preference

**Fig 8:**
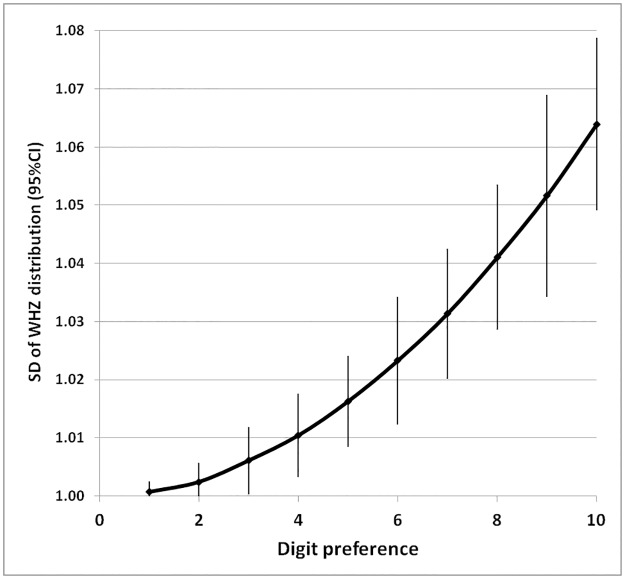
Effect of rounding/digit preference on the SD of WHZ distribution from 50 simulated surveys. The terminal digit of the weights of the children were manipulated using the excel formula: round(weight/n)*n to 0.1Kg where n varied from 1 to 10. The error bars are the 95% confidence intervals.

In the same 50 simulated surveys, rounding was imposed on the true weight of the children as described in the methods section. The effects upon the distribution of the weights-for-height Z-scores are given in Fig 8 and the resultant change in GAM and SAM is shown in Table 3. There was an increase in the SD of the distribution of the Z-scores and the prevalence of GAM and SAM when the data were rounded to simulate digit preference. This increase in the SD and malnutrition prevalence was not as great as the increases seen with the imposition of a random error to the data.

**Table 3:** Effect of rounding/digit preference of weight on the prevalence of GAM and SAM from 50 simulated surveys.

Digit preference	GAM%	SAM%
1	8.03	0.88
2	8.11	0.90
3	8.19	0.92
4	8.37	0.92
5	8.46	1.00
6	8.67	1.03
7	9.01	1.14
8	8.99	1.23
9	9.21	1.32
10	9.87	1.41

The weights of the children were manipulated using the excel formula: round(weight/n)*n to 0.1Kg.

### Effect of measurement error in height, age and MUAC on the resulting distributions and resulting assessment of malnutrition

The effects of random error in the measurement of height, age, weight and height combined and MUAC are shown in Table 4. Each analysis was from a single representative simulated survey with a mean Z-score value of -1.0 Z and replicates of the imposed error term.

**Table 4:** Effect of random error in measurement of height, age, weight and height together and MUAC on the assessment of malnutrition.

SD of random imposed error				replicates	SD	<-2 Z	<-3 Z
Height	Weight	Age	MUAC				
cm	g	mo	mm	#		%	%
**Height-for-age**							
0	-	0	-	50	1.000	15.97	2.13
0.5	-	0	-	50	1.012	16.13	2.25
1.0	-	0	-	50	1.047	16.82	2.61
**1.5**	**-**	**0**	-	50	**1.102**	**18.28**	**3.30**
**2.0**	**-**	**0**	-	50	**1.171**	**19.48**	**4.28**
**2.5**	**-**	**0**	-	50	**1.262**	**21.21**	**5.51**
**Height-for-age**^a^							
0	-	0	-	10	1.001	15.83	2.13
0	-	0.5	-	10	1.031	16.30	2.43
**0**	**-**	**1**	-	10	**1.122**	**17.83**	**3.13**
**0**	**-**	**1.5**	-	10	**1.258**	**19.93**	**4.08**
**0**	**-**	**2**	-	10	**1.453**	**22.25**	**5.88**
**0**	**-**	**2.5**	-	10	**1.686**	**24.37**	**7.88**
**0**	**-**	**3**	-	10	**1.974**	**26.28**	**9.44**
**Weight-for-height**							
0	0	-	-	50	1.004	16.14	2.57
0	50	-	-	50	1.007	16.23	2.64
0	100	-	-	50	1.013	16.30	2.68
0	150	-	-	50	1.024	16.51	2.80
0	200	-	-	50	1.039	16.99	2.99
0	250	-	-	50	1.059	17.42	3.30
0	300	-	-	50	1.081	17.94	3.67
**0**	**350**	-	-	50	**1.106**	**18.53**	**3.99**
**0**	**400**	-	-	50	**1.138**	**19.05**	**4.54**
**0**	**450**	-	-	50	**1.170**	**19.51**	**5.03**
**0**	**500**	-	-	50	**1.204**	**20.33**	**5.60**
**Weight-for-height**							
0	0	-	-	20	1.000	16.14	2.38
0.5	100	-	-	20	1.018	16.81	2.74
1.0	200	-	-	20	1.069	17.80	3.44
**1.5**	**300**	-	-	20	**1.153**	**19.41**	**4.70**
**2.0**	**400**	-	-	20	**1.258**	**21.05**	**6.15**
**2.5**	**500**	-	-	20	**1.378**	**22.83**	**7.85**
**MUAC-for-age**							
-	-	0	0	50	1.000	8.38	1.25
-	-	0	1	50	1.004	8.36	1.30
-	-	0	2	50	1.016	8.58	1.36
-	-	0	3	50	1.036	8.89	1.47
-	-	0	4	50	1.060	9.24	1.65
-	-	0	5	50	1.092	9.92	1.89
-	-	**0**	**6**	50	**1.131**	**10.53**	**2.22**
-	-	**0**	**7**	50	**1.176**	**11.36**	**2.65**
-	-	**0**	**8**	50	**1.226**	**12.05**	**3.09**
-	-	**0**	**9**	50	**1.281**	**13.11**	**3.70**
-	-	**0**	**10**	50	**1.340**	**14.05**	**4.23**

^a^ For this analysis of age variation the random SD of the error was multiplied by age/12

The effect of an error in height measurement of 1.5cm is approximately the same as the effect of 350g error in weight. When there are errors in both weight and in height then the errors are additive and the resultant change in prevalence may be more pronounced. A positive or negative error in both weight and height will tend to cancel each other out (the child is both taller and heavier), whereas a negative error in one with a positive error in the other will magnify the error.

Mother’s recall of age in most communities where surveys for malnutrition are conducted is probably subject to the greatest error and in nearly all surveys there is age “heaping” in the dataset to the nearest 6 and more particularly 12 months. The present analysis confirms that this has the greatest effect upon the calculated results. An error of only 1.5 months, for a 12 month old child (with proportionate errors in older and younger children), can raise the SD from 1.0 to above 1.2. The age is normally only assessed to the nearest month so that the estimate of stunting prevalence is particularly liable to overestimation because of errors of the reported age of the child. Even a random error of 3 months in a 12 month old child can change the assessment of severe stunting (Height-for-age) from 2.1% of children to 9.4% of children. This change in the reported prevalence due to random error is much greater than the expected error which will arise from other problems with the survey such as the sample size and a biased sampling frame, and is greater than the confidence intervals usually reported for HAZ. To this random error must be added any systematic error due to cultural practice of systematically rounding the age up or down, which would have the added effect of moving the mean of the whole distribution up or down. Where ages are rounded up so that the child will be recorded as older than the actual age, there will be an increase in the estimated prevalence of stunting added to the errors due to random error in age estimation. Where ages are rounded down the effect of random error will be mitigated.

In assessment of malnutrition using MUAC it is recommended practice to use the absolute MUAC [32] rather than the WHO MUAC-for-age standards. For this reason the percent of children falling below the internationally agreed cut-off points for the assessment of global and severe malnutrition with an error in MUAC are given in Table 4. It should be noted in this analysis that it is assumed that there is no error in age so that the change in prevalence is entirely due to the change in the accuracy with which the MUAC is measured and thus the error applies equally to absolute MUAC and MUAC-for-age.

When using absolute MUAC, there is only a 10mm difference between normality (125mm) and severe malnutrition (<115mm), and as an acceptable TEM is very difficult to achieve the effect of measurement error is likely to lead to many children being misclassified.

With respect to the precision of the instruments used in anthropometry, errors in weight (300g to bring SD >1.1 with 100g instrument precision) has a less effect than errors in height (1.5cm to bring SD>1.1 with diurnal variation of 0.7cm and difficulties in measurements), MUAC (5mm to bring SD = 1.1, usual TEM of teams of about 4mm) and age (1month to bring SD>1.1 with maximum precision of 1 month in most surveys) is by far the most problematic of all the measurements. With the exception of weight, the precision with which the other parameters can be measured is close to the maximum precision of the instruments used and errors of these measurements may account for most of the errors in survey data.

## Discussion

Our data show that with random error the distribution of each of the parameters that assess the prevalence of malnutrition widens and the numbers of children that fall into the tails of the distribution increases. There is always error of measurement. The observed distribution will always be wider than the true distribution and the prevalence of a condition depending upon a cut-off point defined by a “healthy” range will potentially be systematically overestimated, provided that the “healthy” range has been determined with a less random error than the survey. This appears to be a general phenomenon and our data confirms the theoretical effect of random measurement error and the magnitude of that effect on the quality of the data and consequently the results that will be reported from any survey measuring a continuous variable. This effect does not appear to be general knowledge among those conducting, analysing or interpreting such data. For example, one adviser to the humanitarian community, when asked how a survey could have a low standard deviation for WHZ responded that this could be due to measurement error [33]; such lack of knowledge is common and leads to data being accepted that should be used with circumspection or rejected.

Since the 1930’s measurement errors have been recognised as important and attempts to measure and minimise such errors developed [34–36]. Nevertheless, even though many anthropometric surveys are of poor quality and have been heavily criticised [37,38], measurement error has rarely been invoked or considered as an important cause of misleading results. This is despite the previous recognition that random error can have an effect upon reported prevalence of an anthropometric deficit [39,40], and its incorporation in two recent survey manuals [40–42]. The imprecision with which the instruments are scaled for anthropometry is not thought to have a major effect on the results [8]. In none of the surveys analysed in our survey database [43] have the results of test-retest standardisation to determine the TEM been reported [2]. This is probably because such errors have commonly been thought to be “neutral” and surveyors have considered that such errors can be overcome by simply increasing the sample size. The present analysis shows that this is not the case. Measurement error can inflate the prevalence of a variable to an unacceptable degree and this is not ameliorated by increasing sample size. Consequently the estimates of the burden of diseases and even the definition of normal ranges of biological variables may be misleading. The errors caused by digit preference were found to be the basis for the “Pickering-Platt” controversy about the aetiology of hypertension and other measurement as well as sampling errors often lead to critical misinterpretation of epidemiological data [44].

### Published survey data

The present analysis shows that the effect of measurement error is not trivial, but can change a reported prevalence substantially; amounts that are far greater than the confidence intervals reported in many estimates of malnutrition prevalence. When relatively small scale surveys are conducted by International non-governmental organisations with intensive training and supervision of the staff and ensuring that the enumerators are not overburdened with excess home visits each day, the standard deviations of the WHZ are between 0.8 and 1.2 Z with 80% of them between 0.9 and 1.1 Z [45]. This constancy of the shape of the distribution is maintained even when the prevalence of malnutrition within the population increases to emergency levels. When larger and more complex surveys are considered, for example those included in the WHO database [31] or the DHS (Demographic and Health Surveys) surveys [46] the standard deviation is frequently greater than 1.2 leading to a higher reported prevalence of malnutrition than would be obtained with a focused survey using experienced staff and appropriate data cleaning methods. Although these inflated SDs could in some cases be due to subject heterogeneity it is unlikely that such heterogeneity affects all the populations analysed by Crowe et al. [46]; it is more likely that this effect is due to errors of measurement, recording or data entry.

**Fig 9:**
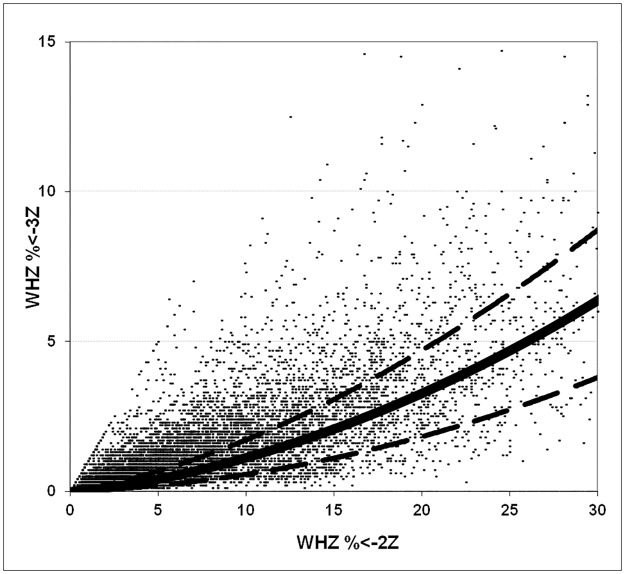
The relationship between GAM and SAM in 9,399 surveys reported in the archival WHO global database on child growth and malnutrition (NCHS standards). If the SD is 1.0 Z then all the surveys’ GAM/SAM ratios should lie upon the solid line, if the SD of the survey is between 0.8 Z and 1.2 Z then the data points should lie between the two dashed lines. Those surveys with points above the upper dashed line have an SD of greater than 1.2Z.

**Fig 10:**
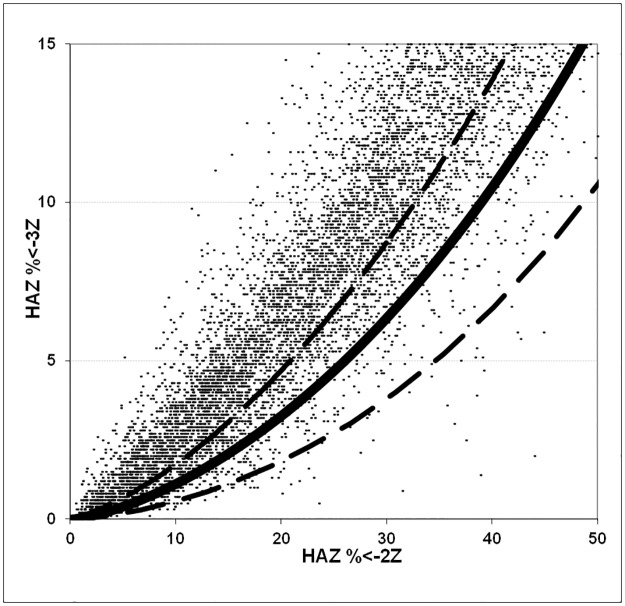
The relationship between Global Stunting (<-2Z HAZ) and Severe Stunting (<-3HAZ) in 10,789 surveys reported in the archival WHO global database (NCHS standards). If the SD is 1.0 Z then all the surveys’ global stunting/Severe stunting ratios should lie upon the solid line, if the SD of the survey is between 0.8 Z and 1.2 Z then the data points should lie between the two dashed lines. Those surveys with points above the upper dashed line have an SD of greater than 1.2Z.

The implications of these findings are quite profound. Fig 9 shows the data from the archival WHO Global Database on Child Growth and Malnutrition using NCHS standards for WHZ (wasting) and Fig 10 shows the same results for HAZ (stunting). Each point represents one survey. The points would all fall on the solid line if the SD of the data was 1.0Z and outside the dashed lines if the SD of the data was >1.2 Z or <0.8. It is clear that for most of the surveys in the database the SD lies above the 1.2Z range and it is likely that in these surveys the prevalence of malnutrition is exaggerated. These data are used to calculate world-wide malnutrition rates and to inform policy and programs at national and international level. However, although publications based upon these data may have exaggerated the prevalence of malnutrition, it should be emphasised that estimates based on prevalence data are also likely to grossly underestimate the true seriousness of malnutrition globally and the numbers of children affected each year. This is because the incidence of malnutrition far exceeds its prevalence. The two effects may thus counterbalance each other, however the extent to which this is the case is unknown. The prevalence data has been used because there are no reliable incidence data.

Table 5 shows the SDs of the anthropometric parameters of individual national surveys carried out in 15 West African countries using the guidelines for DHS and MICS (Multi-Indicator Cluster Survey) survey protocols compared to National Nutrition Surveys (NNS) conducted by national governments using SMART methodology [41, 42]. The DHS and MICS surveys collect an enormous amount of data on many aspects of health, whereas the NNS surveys are focused on anthropometry with rigorous training, supervision, standardisation of each enumerator and plausibility testing of the overall data and of each team; very few additional variables are collected during a SMART survey in order to simplify training, maintain focus and avoid tiring the enumerator, child and family. Although the populations surveyed were the same, the SDs are significantly higher in each of the countries for the DHS & MICS style surveys compared to the National nutrition surveys. This is consistent from country to country and for each indicator. It should be emphasised that the NNS surveys, each with an acceptable SD for WHZ, are also national surveys and therefore are likely to include a heterogeneous mix of subjects. It is not a valid criticism to ascribe the acceptable SD of the NGO’s small scale surveys to lack of subject heterogeneity.

**Table 5:** Comparison of standard deviations from individual National surveys using different survey protocols from West African Countries.

Country	Weight-for-height^d^			Weight-for-age^e^			Height-for-age^f^		
	DHS^a^	MICS^b^	NNS^c^	DHS^a^	MICS^b^	NNS^c^	DHS^a^	MICS^b^	NNS^c^
**Benin**	1.47		**1.09**	1.39		**1.10**	1.87		1.36
**Burkina Faso**	1.42	1.89	**1.07**	1.44	1.67	**1.06**	1.91	1.89	1.21
**CAR**	1.29	**1.14**	**1.15**	1.25	1.24	1.23	1.60	1.56	1.48
**Cameroon**	1.23	1.49	**1.20**	1.20	1.42	1.25	1.51	1.77	1.53
**Chad**	1.48	1.44	**1.08**	1.44	1.51	**1.14**	1.79	2.09	1.44
**Gambia**		1.22	**1.08**		1.20	**1.08**		1.55	1.21
**Guinea**	1.58	1.82	**1.07**	1.52	1.37	**1.19**	2.01	2.05	1.35
**Liberia**	1.34		**1.12**	1.35		**1.16**	1.83		1.46
**Mali**	1.55		**1.10**	1.55		**1.12**	2.10		1.42
**Mauritania**		1.31	**1.07**		1.32	**1.04**		1.76	1.25
**Niger**	2.15	1.29	**1.10**	1.85	1.33	**1.18**	2.47	1.62	1.41
**Nigeria**	1.24	1.31	**1.19**	1.21	1.41	**1.12**	1.57	1.88	1.38
**Senegal**	1.42	1.28	**1.03**	1.44	1.35	**1.08**	1.66	1.69	1.30
**Sierra Leone**	1.53	1.58	**1.12**	1.50	1.42	**1.11**	1.97	1.88	1.31
**Togo**	1.62	**1.05**	**1.03**	1.52	**1.14**	**1.02**	2.08	1.34	**1.19**
**MEAN**	**1.49**	**1.40**	**1.10**	**1.44**	**1.36**	**1.13**	**1.87**	**1.76**	**1.35**
**SD**	**0.24**	**0.26**	**0.05**	**0.17**	**0.14**	**0.07**	**0.26**	**0.22**	**0.11**

The data are from UNICEF West and Central African Regional Office, Unpublished report, “Report on the Quality of Anthropometric Data on Children’s Height and Weight in 100 Health and Nutrition Surveys in the West Central Africa Region”. CAR = Central African Republic. The increasing density of pink coloured shading shows results that are over 1.2, 1.5 and 1.8Z respectively.

^a^ Demographic and Health Survey

^b^ Multi-Indicator Cluster Survey

^c^ National Nutrition Survey

^d^ WHZ—highly significant ANOVA df 2,37, F = 15.0, p<0.000; there is no difference between DHS and MICS p = 0.39

^e^ WAZ -highly significant ANOVA df 2,37, F = 21.7, P<0.000; there is no difference between DHS and MICS p = 0.27

^f^ HAZ—highly significant ANOVA df 2,37, F = 25.8, p<0.000; there is no difference between DHS and MICS, p = 0.24

Table 6 shows the data from 100 DHS, MICS and NNS surveys. These data include surveys from additional countries and years from those shown in Table 5. The prevalence of GAM and SAM derived from the mean of the observed SDs and also the prevalence that would have been reported had the SD been 1.0Z or 1.1Z are shown. For WHZ deficits the DHS and MICS surveys give about twice the prevalence than would have been reported if the surveys’ SDs were within the expected “good” range; for severe malnutrition the DHS and MICS surveys are close to ten times the expected values. With the NNS surveys the prevalences are within the acceptable range for WHZ. Weight-for-age and height-for-age values are higher which are probably due to difficulties in age determination; the discrepancy is much greater with the DHS and MICS surveys than the NNS surveys.

**Table 6:** The GAM and SAM from 100 West African Surveys computed with the average observed SDs and prevalence that would obtain if the SD had been either 1.0Z or 1.1Z.

	N	Mean Z	SD	SD obs	SD = 1.1	SD = 1	SD obs	SD = 1.1	SD = 1
**Weight-for-height**				Moderate (<-2Z)			Severe (<-3Z)		
DHS^a^	45	-0.35	1.44	**12.6**	6.7	5.0	**3.3**	0.8	0.4
MICS^b^	28	-0.26	1.45	**11.4**	5.6	4.1	**2.9**	0.6	0.3
NNS^c^	27	-0.54	1.11	**9.3**	9.2	7.2	**1.3**	1.3	0.7
**Weight-for-age**				Moderate (<-2Z)			Severe (<-3Z)		
DHS^a^	45	-1.08	1.39	**25.3**	20.0	17.8	**8.3**	4.0	2.7
MICS^b^	28	-1.06	1.40	**25.1**	19.7	17.4	**8.3**	3.9	2.6
NNS^c^	27	-1.14	1.13	**22.3**	21.7	19.5	**5.0**	4.5	3.1
**Height-for-age**				Moderate (<-2Z)			Severe (<-3Z)		
DHS^a^	45	-1.40	1.80	**37.0**	29.3	27.5	**18.7**	7.3	5.5
MICS^b^	28	-1.46	1.82	**38.4**	31.3	29.6	**20.0**	8.1	6.2
NNS^c^	27	-1.35	1.36	**31.7**	27.7	25.8	**11.3**	6.7	4.9

Data are taken from: UNICEF West and Central African Regional Office, Unpublished report, “Report on the Quality of Anthropometric Data on Children’s Height and Weight in 100 Health and Nutrition Surveys in the West Central Africa Region”, 2014. The prevalence of global (<-2Z) and acute (<-3Z) wasting, underweight and stunting presented are calculated using the normal density function (“normdist”) in excel. Obs = observed mean survey SD.

^a^ Demographic and Health Survey

^b^ Multi-Indicator cluster Survey

^c^ National Nutrition Survey (using SMART methodology)

**Fig 11:**
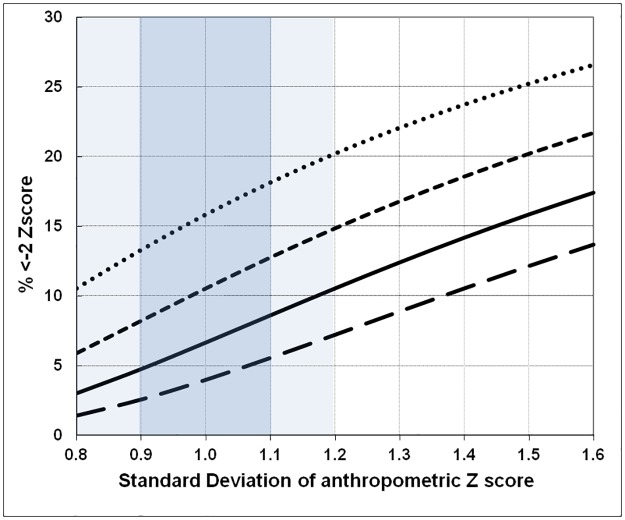
The percent of children with moderate malnutrition (<-2.0 Z) with a change in the SD of a survey, based upon a Gaussian distribution. The mean Z of the distributions from top to bottom are -1.0 Z, -0.75 Z, -0.5 Z and -0.25 Z. The area representing an “acceptable” survey is given in light blue and a “good” survey in heavier blue.

**Fig 12:**
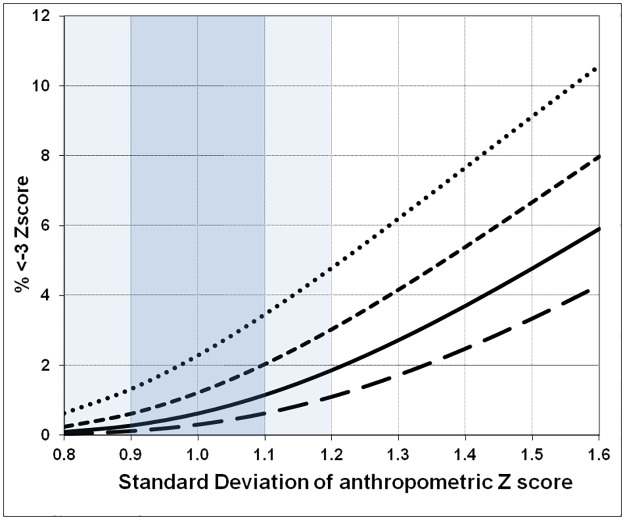
The percent of children with severe malnutrition (<-3.0 Z) with a change in the SD of a survey, based upon a Gaussian distribution. The mean Z of the distributions from top to bottom are -1.0 Z, -0.75 Z, -0.5 Z and -0.25 Z. The area representing an “acceptable” survey is given in light blue and a “good” survey in heavier blue.

The effect of a change in the SD from 0.8 to 1.6 upon the prevalence of Global malnutrition (wasting, stunting, MUAC-for-age) defined as a z-score of <-2.0Z, with population means from -0.25Z to -1.0Z, is shown in Fig 11. Even within the “acceptable” range of survey results, 0.8 to 1.2, and a “good” range of 0.9 to 1.1, there are potential major changes in the prevalence computed from the raw survey data. Thus, with a mean Z-score of -0.75 Z an SD of 0.9 corresponds to a prevalence of 8.2% and an SD of 1.1 to a prevalence of 12.8%. Even within the “acceptable” range, the prevalence changes from 5.9% to 14.9%, a difference that moves the population from almost normal to a severe emergency situation. The corresponding change in severe malnutrition (<-3.0Z) is shown in Fig 12. With a population mean Z-score of -1.0Z, an SD of 0.9 corresponds to a SAM rate of 1.3% whereas an SD of 1.1 gives a SAM rate of 3.5%—when the distribution is even more spread the prevalence rises alarmingly—at an SD of 1.6 the severe malnutrition rate exceeds 10%. This demonstrates the dramatic effect that measurement error can exert on reported results. Even within the acceptable range of survey data the SAM prevalence can vary by up to five fold. In our opinion reports from surveys with an SD of more than 1.2 are unreliable. The problems arise particularly with the estimate of SAM prevalence. These data are used to estimate the numbers of malnourished children that need to be treated and therefore the scale of the intervention that needs to be planned in terms of obtaining funds, procurement of supplies and deployment of trained staff.

An analysis of DHS and MICS shows elevated SD values with all of the mean SDs outside the acceptable range; none of mean SDs for any of the surveys was less than 1.0Z. In agreement with the data from West Africa, the 5th and 95th centiles of the SDs of 51 recent DHS surveys were HAZ 1.35–1.95; WAZ 1.17–1.46, and WHZ 1.08–1.50 [47]. Mei & Grummer-Strawn conclude that they “concur with the WHO assertion that SD is in a relatively small range”. Figs 11 and 12 demonstrate that such “small” differences are not of little consequence but encompass potentially massive errors in the reported prevalence, particularly for severe malnutrition. Analysis of the NHANES data from the USA National surveys, which were enormous surveys of an undoubtedly heterogeneous population shows that despite the ethnic diversity and geographically large scale of these surveys, the SDs were all close to 1.0 Z. The DHS surveys are extensively used for research and direct funding decisions. Short et al. [48] identified 1117 peer reviewed publications based on these data and reported that the number based on the use of DHS data were highly correlated with trends in funding for health by the Government of the USA and globally.

It is clear that even small changes in the SD of the distribution can have a profound effect upon the results obtained. Within the good range of SDs most values are likely to be reasonably reliable for moderate malnutrition; however, the effects of measurement error cannot be dismissed as trivial. These numbers are important. The prevalence of GAM and SAM are used internationally to declare an emergency and mobilise funds and other resources to provide humanitarian relief. Severe malnutrition is a much more lethal condition and is relatively expensive to treat; accurate estimation of the burden is critical to provide the products, staff and facilities to treat all the children with severe acute malnutrition. Overestimation is wasteful and diverts scarce resources from where they could more usefully be deployed.

### Should we be concerned about measurement error?

The whole of public health depends upon having reliable data. The question then arises as to whether random error is simply of theoretical interest or is of practical importance, and whether the differences are trivial or lead to major changes in perception, public health policy, the distribution of resources and the relative priority given to different countries and emergencies. We are aware of several surveys that clearly were misleading.

- A university in Asia did a large survey using its students in the capital city. They reported a SAM rate of 6% which was not in agreement with the Government’s survey which gave a prevalence of 1.3%. The SD of the University’s survey was 2.1 Z. Recalculation of the raw results using SMART flags and an SD of 1.0 gave a SAM rate of 1% which is within the confidence intervals of the national survey.
- In an East African country, two agencies did surveys in the same area from the same population at almost the same time. The International Agency reported a GAM rate of 20% (SD not reported) whereas the non-governmental organisation (NGO) reported 6% (SD 1.13).
- A large and complex survey was undertaken in an African country which gave a GAM rate of 19.8%; the WHZ SD was 1.59Z. As the result was questioned due to few malnourished children being admitted for treatment an NGO was asked to do a validation survey in the worst area of the original survey. They found a GAM rate of 4.8% with an SD of 1.03Z.
- In an East African country a survey was used to estimate that there were about 5,000 severely malnourished children were present in one large district needing immediate care. Mobile teams were established to scour the district for malnourished children and give relief. Over a 5 month period only 650 children were discovered with SAM in the whole district.

None of these cases were reported by the agencies involved. Each was caused by a poorly conducted, analysed, cleaned and reported anthropometric survey.

Such problems are very rarely reported because of fears that they will affect careers, organisations’ reputations and funding. These surveys also influence National Governments and are used to demonstrate whether or not a country is meeting, or failing to meet, such milestones as the Millennium Development Goals (MDGs); this can impact their access to bi-lateral funds and is politically sensitive. In fact, it is possible that some MDGs could be thought to be met, with no change in the actual situation on the ground, simply from an improvement in the quality of surveys and a reduction of random errors of measurement as well as correction of other survey problems [37]. With a move towards regarding stunting in height (height-for-age) as a critical parameter of national nutritional health this becomes particularly problematic as the assessment of age is subject to relatively large errors in countries without accurate birth registration compared with measurements of weight, height and MUAC. By suppressing the reports, lessons are not learned so that the mistakes are repeated.

Surveys are expensive and difficult to conduct with sufficient rigor to give reliable results. Teams are often recruited without any previous experience, are trained for a few days, often only in the class-room, but do not have to undergo any rigorous field evaluation or standardisation tests; they are often relatives or friends of the staff involved in organising the survey. The problems of conducting a reliable survey are many. They include problems of assessing population size, the topography, inaccessible areas, insecurity and physical threats to the teams, population movement, nomadic populations and sampling in urban areas and slums. If these difficulties are overcome and funds expended it is inexcusable to produce completely erroneous data because having arrived at the correct household the measurements were not taken with sufficient precision and accuracy to give valid estimates of the parameters of interest.

There are many questions that policy makers require answered in order to organise services and use resources most efficiently. A survey provides the data to address those questions; it is the sensory nervous system of the government upon which the motor system depends. Calls to involve professional experts and having extensive training have largely gone unheeded [49]. We would advocate for Governments and Agencies to have permanent full-time survey teams employed that would undertake a whole range of different surveys in their populations, each focused on answering different sets of questions for which the various ministries require data. All technical staff improve with familiarity and practice, as the improvements of the technical errors from one survey to the next show [50]. The practice of recruiting large numbers of enumerators, most of whom have not been involved frequently in previous surveys, in multiple teams for several weeks or months at irregular intervals is bound to give erratic results, no matter how well they are trained over a short period of time. We consider such practice as unacceptable. The DHS’s commissioned evaluation states that “highly significant variation across interviewers is found in most surveys” [51].

### Implications for data cleaning

The question then arises as to how best to clean and analyse raw data from the field that is subject to random measurement, rounding, recording and data-entry error. The standard way recommended by WHO is only to exclude data considered to be biologically impossible and incompatible with life. This creates a very wide gate for all results that could possibly occur biologically to be included, even if they are in error. A few extreme values may be true but others are most likely to be errors. One major problem with identifying children with extreme values is the relatively small number of such subjects that are found in a survey with a manageable sample size. Thus, if we have 900 subjects in a survey and the true prevalence of SAM is 1%, then there are in fact only 9 actual children in the survey designated as having SAM. If in taking the 900 measurements of height or weight, 9 or more children’s data contain errors that would lead the children to be in the extreme tail of the distribution but not yet biologically impossible, those 9 children will also be classified as SAM, and effectively double the reported prevalence. The usual objective of a survey is to determine the prevalence of malnutrition in a defined population by selecting a representative sample of that population. Rather than include all biologically plausible values, we would want to exclude from analysis those measurements that are in error. Realistically this means excluding values that are much more likely to be errors than true values. This can be done statistically. If the population approximates a normal distribution then 99.8% of the children that are truly representative of the population should lie within ± 3.1 standard deviations of the mean of the population. In other words if there is a survey of 1000 children, on average, there will be 1 child with a Z score below and one child with a Z-score above the range of ± 3.1 SD from the mean of that population whose result is correct and that child is appropriately classified; the other children outside this range are much more likely to be errors of measurement, recording or data-entry than true values and should therefore be excluded from the analysis. More contentiously, even if more than one child’s measurements are accurate it could be argued that this child is unlikely to be representative of the population. If, for example, a child from a rich country with a weight for height of +2.1Z is visiting relatives in an impoverished country with a population mean weight-for-height of -1.0 Z and is by hazard included in the survey, then that child would not be representative of the population; alternatively, a particular child may have an inborn error of metabolism or a very rare disease which renders him unrepresentative of the population that is being surveyed.

For these reasons it is strongly recommended that the “flags” used to clean the data should be set in relationship to the mean of the population under consideration not in relationship to values that are incompatible with life. When the different types of flags are compared the prevalence of malnutrition reported is less when using deviation from the population mean than using extreme values; the higher the standard deviation of the survey population’s Z score the larger the discrepancy between the two methods of cleaning the data [46]; this is a consequence of both the theory and the analysis presented herein.

If a survey is thought to contain many errors of measurement, indicated by an excessive standard deviation, the present analysis suggests that the survey results should be rejected. However, this would be excessively wasteful of time and resources and potentially delay urgent action until reliable data is obtained. In theory, if the distribution is (approximately) Gaussian, there is a trivial change in the mean value of the data with random measurement, digit preference or rounding error. In these circumstances we can calculate the prevalence of GAM and SAM from the cumulative normal distribution function (probit) [3]; an SD range of 0.9 to 1.1 could be presented where the SD is still excessive after excluding flagged data. In this way surveys can be “rescued”. However, inferences should only be drawn with great caution because the data have been taken without sufficient training or care; a rapid, small-scale “validation” survey should be conducted and reported along with the “rescued” main survey. We would suggest that the confidence intervals which are calculated conventionally from the relative proportions above and below the cut-off should always be compared with calculated estimates, and if possible a correction made for the technical errors of the measurements. Nevertheless, the report should make it clear that the data need to be interpreted with circumspection.

### Reporting

Apart from reporting the results of the survey, all reports should also include the following information: the results of standardisation tests: the numbers of subjects excluded with the various flagging methods; the age and sex distribution of the subjects; the distribution of the data; and, the mean and SD of the parametric data collected in the survey. The raw uncleaned data should be made available for external evaluation and for research purposes.

## Conclusion

Measurement error is always present and will inflate the number of cases below a cut-off point in the tail of a distribution. With anthropometry even relatively small errors can increase the reported prevalence of malnutrition substantially. This has implications for prioritising resources to areas with the highest prevalence and for how the data is analysed and reported.

Measurement error can also misclassify individual children that are referred for treatment, but this is likely to lead to more children being included than excluded and therefore does not represent a threat to individuals screened for treatment programs.

## Abbreviations

GAM: global acute malnutrition
HAZ: height-for-age Z-score
LMS: lambda-mu-sigma, statistical parameters in constructing density curves
MUAC: mid-upper arm circumference
MUACage: MUAC-for-age
SAM: severe acute malnutrition
SD: standard deviation
SD^2^: observed variance
TEM: technical error of the measurement
TEM^2^: variance of the TEM
WAZ: weight-for-age Z-score
WHZ: weight-for-height Z-score
